# Cognitive restraint directed at carbohydrates in individuals on low-carb diet with binge eating: the role of guilt about food cravings

**DOI:** 10.31744/einstein_journal/2021AO5599

**Published:** 2021-04-01

**Authors:** Jônatas de Oliveira, Maíra Stivaleti Colombarolli, Leandro Silva Figueredo, Táki Athanássios Cordás

**Affiliations:** 1 Faculdade de Medicina Universidade de São Paulo São PauloSP Brazil Faculdade de Medicina, Universidade de São Paulo, São Paulo, SP, Brazil.; 2 Universidade de São Paulo Ribeirão PretoSP Brazil Universidade de São Paulo, Ribeirão Preto, SP, Brazil.; 3 Beneficência Portuguesa de São Paulo São PauloSP Brazil Beneficência Portuguesa de São Paulo, São Paulo, SP, Brazil.

**Keywords:** Binge-eating disorder, Diet, carbohydrate-restricted, Cognitive restraint

## Abstract

**Objective:**

To evaluate whether the carbohydrate-restricted diet leads to higher levels of food cravings in individuals with binge eating.

**Methods:**

A total of 146 individuals with binge eating participated in the Low-Carb Diet Group (n=48) and Control Group (n=98). The Binge Eating Scale, Hay’s questionnaire, Food Cravings Questionnaire - Trait and State, Cognitive restraint subscale and its adapted version for the cognitive restraint toward carbohydrates, were used as measures. Parametric tests were used for comparison between groups (Student’s *t* test), and Pearson’s correlation test to verify correlations between variables of interest.

**Results:**

No differences were found between groups with and without diet concerning the level of binge eating or food craving total score. The differences found were the higher levels of cognitive restraint (p=0.01), cognitive restraint for carbohydrates (p=0.01) and subscales of ‘guilt about food craving’ (p=0.04) in the Low-Carb Diet Group.

**Conclusion:**

Individuals with binge eating and a history of low-carb diet have greater cognitive restraint toward carbohydrates and association with altered eating attitudes (guilt about food craving).

## INTRODUCTION

The practice of restrictive diets has been popularly employed for weight loss, including motivations such as the need for a change in lifestyle, treatment of chronic diseases, and beauty and aesthetics.^(1)^The use of some practices without professional guidance involves thinking aimed at food control, related to a “cognitive restraint” (*e.g.*, “I do not eat some foods because they make me fat”).^(2)^The behavioral strategies of losing weight triggered by a cognitive restraint, such as skip meals, fast, portion control, and calorie counting, are associated with negative attitudes relative to the body.^(1)^

The decrease in consumption of carbohydrates in the diet has promoted a series of nomenclatures. Diets in which this consumption varies within the range of 30g to 130g per day are considered low-carb.^(3)^ In this setting, there are several possible dietary variations, such as the control of energy content aiming at a negative energy balance, making a hypocaloric low-carb diet. It is indicated for the treatment of obesity, since it causes less release of insulin and a greater release of glucagon, facilitating fat oxidation and sparing lean mass.^(3)^

When the decrease of carbohydrates is severe (quantity equal to or less than 20g a day), a state of ketosis is induced, and the diet is included in the nomenclature of a ketogenic diet.^(3)^One example is the Atkins diet, aiming at consumption lower than 20g of carbohydrates per day, during the first two weeks.^(3)^

Regarding the appearance of an intense desire for food resulting from this type of restriction, Castro et al., demonstrated a very low-calorie ketogenic diet (supervised prescription) did not cause increased food craving.^(4)^ The same was described by Anguah et al., who verified a decrease of food craving and an increase by 102% in cognitive restraint,^(5)^ when using a ketogenic diet with proportions of 14% of carbohydrates, 58% of fat and 28% of protein. In another randomized clinical trial using a low-carb diet, individuals who lost more weight had an increased desire for caloric foods in the sixth month, but gave in to desires less frequently.^(6)^

Food craving is defined as a feeling of strong desire that involves sensation of urgency, with negative affection, and a series of thoughts directed towards consuming anticipation, always for the consumption of a specific food.^(7)^ This condition includes cognitive, emotional and neurophysiologic aspects, besides being influenced by the environment and by the availability of food.^(8)^ The increased craving is a characteristic commonly observed in individuals that present episodes of binge eating (BE), characterized by the objective consumption of a large amount of food occurring in a short period of time, under the sensation of loss of control.^(9)^

Considering the practice of carbohydrate restriction, and how it can influence the increase of craving, the literature presents limited evidence on this relation in individuals with characteristics of BE behavior.

## OBJECTIVE

Assess whether the practice of a carbohydrate-restricted diet is related to higher levels of food cravings in individuals with binge eating.

## METHODS

### Participants

This study is characterized by a cross-sectional design, performed with a sample of 853 students from the *Universidade de São Paulo* (USP). Of these, 146 participants were selected for this study, in which 124 (84.9%) were female. For the inclusion criteria, individuals of both sexes were selected whose scores on the Binge Eating Scale (BES) were indicative of the presence of BE (score >17). Respondents who declared they were not from the university students were excluded from the study. In addition, undergraduate Nutrition students were disregarded for knowledge of nutritional adequacy and for the high prevalence of risk for eating disorders. In this study, those who reported the presence of some inappropriate compensatory behaviors (self-induced vomiting, laxatives, and diuretics) or who presented with low weight, according to the body mass index (BMI <18.5kg/m^2^), were also excluded.

### Instruments

#### Binge Eating Scale (BES)

BES has been amply used to check the presence and severity of BE symptoms.^(10)^ It has statements in Likert format, and measures the frequency of BE symptoms. The version used was translated and validated in Brazil.^(11)^ The interpretation of scores is based on cut-off scores, classifying individuals regarding the presence and severity of BE. For example, absence of binge eating if ≤17 points; moderate binge eating if between 18 and 26 points, and severe BE if ≥27 points. In a study to verify the sensitivity of the scale to the presence of binge eating disorder (BED), the cut-off score of 17 points was compared with the Structured Clinical Interview (SCID), showing a sensitivity of 97.9%, the test-retest reliability according to the Kappa coefficient (0.65), and the weighted Kappa of 0.66, with Cronbach´s alpha of 0.89, demonstrating the adequacy of their psychometric characteristics in the Brazilian population.^(12)^

#### Hay’s Questionnaire

Developed by Phillipa Hay, this questionnaire evaluates the frequency of inappropriate eating behaviors, such as BE, compensatory methods, and restrictive diet practice in the last three months.^(13)^In this study, the adapted version for Brazilian Portuguese was used, which showed acceptable reliability indicators, with a Kappa value of 0.92 in the validation study of this version. Specifically, questions evaluating the presence of inappropriate compensatory methods and the frequency of BE episodes were used.^(14)^

#### Cognitive restraint subscale of the Three Factor Eating Questionnaire

The cognitive restraint subscale is related to food restriction with the objective of modifying weight or body shape,^(2)^ and was proposed in its reduced version with 21 questions.^(15)^ In Brazil, it was adapted to Portuguese^(16)^ and later validated.^(17)^ The scale is organized in Likert format of four points, for items from one to twenty, and of eight points for question 21. Regarding the interpretation of scores, the higher values indicate higher levels of cognitive restraint. In the present sample, the reliability indicators of this instrument were considered adequate, with Cronbach´s alpha of 0.83.

#### Cognitive Restraint Subscale adapted for carbohydrates

The same cognitive restraint subscale of the Three Factor Eating Questionnaire^(15,16)^ was adapted, with the inclusion of terms specifically related to carbohydrate restriction. This adaptation was communicated and previously approved by the author of the scale, Dr. Jan Karlsson, from Örebro University, Sweden, to meet the objectives of this study. In the questionnaire header, participants were instructed about some foods that are carbohydrate sources. The score and transformation in the total score was maintained as in the original version, and no cut-off scores were used. The highest values indicated the highest level of “targeted carbohydrate restriction”. Cronbach’s alpha calculated in this sample for cognitive restraint scale adapted for carbohydrates was 0.84, demonstrating adequate reliability rates. The original items and their adaptation can be found in Appendix 1.

#### Food Cravings Questionnaire - Trait and State

Cepeda-Benito et al., developed the Food Cravings Questionnaire - Trait (FCQ-T) and the Food Cravings Questionnaire - State (FCQ-S), which combine two instruments evaluating different aspects of food craving: one assesses craving as a constitutional element (FCQ-T), and the other as a transitory element (FCQ-S).^(8)^ In this study, the two versions adapted to Brazilian Portuguese were used, with satisfactory results in the analyses conducted.^(18)^ The Cronbach’s alpha calculated in this sample was 0.94 for FCQ-T and 0.88 for FCQ-SE, demonstrating satisfactory reliability indicators.

#### Food frequency questionnaire

To evaluate the consumption of foods that are rich of carbohydrates, questions were used from a food frequency questionnaire for the last three months. This questionnaire is composed of a seven-point Likert scale, covering frequencies from “rarely or never” to “2 or 3 times a week”. Six types of foods were selected (tin loaf, French roll, chocolate, white rice, pasta, and salty crackers).^(19)^

#### Question to identify the low-carb diet

To identify carbohydrate restriction diet in the last three months, a following question was employed: In the last three months, have you tried to be on a low-carb diet, avoiding foods thar are sources of carbohydrate? (The answer options were “yes”, “no” and “I do not know what a low-carb diet is”).

## Procedures

### Data collection

The recruitment of participants was done through dissemination in specific social networking groups (Facebook^®^) and through institutional e-mails. Those who received e-mails were also invited to share the access link of the online questionnaire on social networks, with other university students from the same institution. The data were collected between July 2018 and March 2019. All types of information were collected using structured questions and evaluation scales. Information on height and weight was self-reported. All the ethical precepts of research on human beings provided by the National Research Council were met, having been duly registered and approved by the institution in charge (approval number: 2.695.532; CAAE: 88846718.7.0000.0065).

## Data analysis

Initially, the reliability indexes of the scales in the studied sample were calculated using Cronbach’s alpha. Then, the descriptive analyses of the data were carried out to identify the measures of central tendency of the numerical variables (means and standard deviation - SD), and absolute and relative frequency for the categorical variables. The normality of the variables was analyzed considering the asymmetry and kurtosis parameters up to 2.0 and 7.0, respectively, to verify distortions in the data distribution, according to criteria suggested by Kim.^(20)^ After verifying the normality in the distribution of the variables in both groups, the parametric tests were continued to compare the mean results between groups (Student’s *t* test) and verify correlations between the variables of interest (Pearson’s correlation). The effect size (Cohen’s d) was also calculated as the difference of the means between groups. For the categorical variables, the distribution analysis and comparison between groups was done using the χ^2^ test. The analyses were conducted by the SPSS software, version 25.

## RESULTS

Considering the sample of individuals with binge eating (n=146), 48 participants who went on a carbohydrate restriction diet (Low-Carb Diet Group), and 98 who did not go on this diet (Control Group) were identified. The mean age of the Control Group was 22.1 years (SD±3.25), while in the Low-Carb Diet Group, the mean age was 22.0 years (SD±3.1), with no significant differences between the groups (*t*=0.77; p=0.78). As to BMI, both groups were statistically comparable, with a mean BMI of 26.5kg/m^2^ (SD±5.4) for the Control Group and 26.5kg/m^2^ (SD±4.5) for the Low-Carb Diet Group, with no differences between them (*t*=-0.04; p=0.96). Additionally, table 1 displays the data on health characteristics, life habits, and clinical and psychiatric diagnoses for both groups.

**Table 1:** Distribution of the participants according to diet

	Control Group (n=98) n (%)	Low-Carb Diet Group (n=48) n (%)
Sex		
Male	18 (18.4)	4 (8.3)
Female	80 (81.6)	44 (91.7)
BMI classification		
Eutrophic	47 (48)	26 (54.2)
Overweight	28 (28.6)	12 (25)
Obesity	23 (23.5)	10 (20.8)
Smoking, cigarettes per day		
1-3	4 (4.1)	1 (2.1)
4-5	5 (5.1)	2 (4.2)
>10	2 (2.0)	1 (2.1)
Do not smoke	87 (88.8)	44 (91.7)
Alcoholic drink consumption, times per month		
1-3	44 (44.9)	18 (37.5)
1	9 (9.2)	5 (10.4)
2	11 (11.2)	7 (14.6)
3	4 (4.1)	3 (6.3)
Does not consume alcohol	30 (30.6)	15 (31.3)
Type of feeding		
Omnivore (consumes meat)	83 (84.7)	45 (93.8)
Vegan	2 (2.0)	0
Vegetarian	13 (13.3)	3 (6.3)
Chronic diseases		
Hypercholesterolemia	4 (4.1)	2 (4.2)
Hypertension	1 (1.0)	1 (2.1)
No chronic diseases	93 (94.9)	45 (93.8)
Diagnosis of ED		
Anorexia nervosa	2 (2.0)	3 (6.3)
BE disorder	12 (12.2)	6 (12.5)
No diagnosis of ED	84 (85.7)	39 (81.3)
Psychiatric diagnosis		
Depression	5 (5.1)	4 (8.3)
Bipolar disorder	2 (2.0)	1 (2.1)
Anxiety disorder	20 (20.4)	3 (6.3)
Anxiety and depression disorder	27 (27.5)	15 (31.2)
No psychiatric diagnosis	44 (44.9)	25 (52.1)
Episodes of BE		
Two or more times a week	24 (24.5)	8 (16.7)
Once a week	31 (31.6)	20 (41.7)
Less than once a week	33 (33.7)	19 (39.6)
No episodes	10 (10.2)	1 (2.1)

BMI: body mass index; ED: eating disorder; BE: binge eating.

To identify the relation between diet with carbohydrate restriction and the presence of BE symptoms, comparative analyses were made using this variable as a criterion (Table 2). No differences were found between the mean scores reported for BE symptoms, nor in the mean scores of the FCQ-T and the FCQ-S. Among the differences found, higher levels of cognitive restraint, and cognitive restraint for carbohydrates, were observed in the Low-Carb Diet Group, this difference was significant and the effect size considered large (d=1.02 and 1.15, respectively). The Low-Carb Diet Group also presented with higher levels of guilt over food craving, according to the FCQ-T (p=0.04; d=0.36), compared to individuals in the Control Group, with the difference presenting a moderate effect size.

**Table 2:** Comparisons of the mean scores in the evaluation instruments, according to diet with carbohydrate restriction

	Control Group (n=98)		Low-Carb Diet Group (n=48)		t*	df	p value	d^†^
	Mean	Standard deviation	Mean	Standard deviation				
Binge eating	23.74	4.84	23.35	5.27	0.43	144	0.66	0.08
Scale of cognitive restraint	13.31	4.38	17.75	4.35	5.77	144	0.01	1.02
Cognitive restraint of carbohydrates	11.74	3.74	16.00	3.70	6.50	144	0.01	1.15
FCQ-T	160.75	29.59	164.58	27.06	0.75	144	0.45	0.13
Intentions and plans to eat	13.56	3.03	13.52	3.38	0.07	144	0.94	0.01
Anticipation of positive reinforcement	21.06	4.76	20.98	4.95	0.09	144	0.92	0.02
Anticipation of the relief of negative feelings	12.15	3.45	12.08	3.71	0.11	144	0.91	0.02
Lack of control	23.25	6.29	24.38	5.83	1.04	144	0.29	0.18
Thoughts or concerns	24.88	7.40	26.08	6.77	0.95	144	0.34	0.17
Desire as a physiological state	17.79	3.79	17.54	4.09	0.35	144	0.72	0.06
Emotions present in desires	18.38	4.34	18.17	4.62	0.27	144	0.78	0.05
Triggers	17.64	4.01	18.40	3.71	1.09	144	0.27	0.19
Guilt for wishes or for having given in to them	12.04	4.08	13.44	3.59	2.01	144	0.04	0.36
FCQ-S	49.15	10.55	49.33	11.30	0.09	144	0.92	0.02
Craving	10.60	3.20	10.33	2.98	0.48	144	0.62	0.09
Anticipation of positive reinforcement	10.06	3.05	10.23	2.58	0.32	144	0.74	0.06
Anticipation of the relief of negative feelings	9.38	2.73	9.75	3.26	0.72	144	0.47	0.13
Lack of control	10.03	2.70	9.73	2.93	0.61	144	0.53	0.11
Desire as a physiological state	9.08	3.05	9.29	3.34	0.37	144	0.70	0.07

* Student’s *t* test statistics; ^†^ effect size expressed by Cohen d. df: degrees of freedom; FCQ-T: Food Craving Questionnaire - Trait; FCQ-S: Food Craving Questionnaire - State.

The groups were also compared according to the BMI classification, noting that the presence of people with eutrophic weight, overweight, or obesity did not differ between groups. In the Low-Carb Diet Group, 26 were eutrophic, and 22 were overweight or have obesity. In the Control Group, 48 were in the normal weight range, while 47 were overweight or have obesity (χ^2^ 0.16; p=0.68). Additionally, the possible relations between the variables of BMI, BE, cognitive restraint, cravings, and carbohydrate restriction were also analyzed. Furthermore, we observed the relations between these variables and the consumption of food considered rich in carbohydrates. The results are expressed on table 3.

**Table 3:** Pearson’s correlation of mean scores of the variables body mass index, evaluation instruments, and frequency of consumption of carbohydrate-rich foods (n=146)

	BMI	Binge eating	Cognitive restraint	FCQ-T	FCQ-S	Cognitive restraint of carbohydrates
BMI						
Binge eating	0.20^*^					
Cognitive restraint	-0.04	-0.08				
FCQ-T	0.11	0.47^†^	-0.03			
FCQ-S	0.10	0.25^†^	-0.11	0.66^†^		
Cognitive restraint of carbohydrates	-0.02	-0.06	0.90^†^	0.02	-0.13	
Chocolate	-0.07	-0.04	-0.12	0.02	0.05	-0.13
Tin loaf	-0.03	0.02	0.01	-0.03	-0.12	-0.06
Rice	0.03	0.05	-0.33^†^	-0.07	0.03	-0.34^†^
French roll	0.12	0.26^†^	-0.13	0.13	0.05	-0.14
Pasta	-0.04	0.04	-0.14	0.17^*^	0.19^*^	-0.21^†^
Cookie/cracker	0.21^**^	-0.07	-0.06	-0.11	-0.11	-0.09

* p<0.05; ^†^ p<0.01. FCQ-T:

Food Cravings Questionnaire - Trait; FCQ-S: Food Cravings Questionnaire - State; BMI: body mass index.

The analyses indicated a positive relation between BMI and BE symptoms (r=0.20) and the consumption of cookies/crackers (r=0.21), and these magnitudes were considered weak. BE was related to the weak to moderate increase of desires on both scales of the FCQ-T (r*=*0.47) and FCQ-S (0.25). Cognitive restraint was directly and strongly related to the greatest cognitive restraint of carbohydrates (r=0.90), and moderately related to a lower consumption of rice (r=-0.33). The cognitive restraint of carbohydrates, on the other hand, was related to a lower consumption of rice (r=-0.34) and pasta (r=-0.21), with the latter showing a weak association.

## DISCUSSION

In this study, some aspects of eating behavior were compared in individuals with BE who reported having made, or not, restriction of carbohydrates (the so-called low-carb diet) over the previous three months. The comparison demonstrated food craving data were not greater in those who reported going on diet. The differences found were the greatest levels of cognitive restraint and cognitive restraint for carbohydrates in those on the low-carb diet.

### Cognitive restraint and relation with unsupervised diet

When evaluating the cognitive restriction directed at carbohydrates, it was possible to observe that a lower consumption of rice and pasta was directly related to the profile of restrictive thoughts. Cognitive restraint can be related to disordered eating attitudes, as in the example of a subscale of cognitive restraint, (“I do not eat some foods because they make me fat”). In individuals with BE, these attitudes promote troubled eating, because they disregard the context and frequency in which “some foods” could contribute to weight gain.^(21)^

Thus, carbohydrate restriction should be discussed as a possibility of intervention if it occurs under specialized care, for a determined time, with adaptation phases and behavioral evaluations.^(4,5)^Nevertheless, the prevalence of unsupervised practice of restrictive diets is increasing.^(22-25)^ Hume et al., demonstrated that individuals who went on successful diets reported lower consumption of carbohydrates, higher dietary restriction, higher protein intake, and more vigorous physical activity.^(26)^

In this study, half of the group that went on a diet with carbohydrate restriction reported eutrophic weight, leaving doubt about the motivation for diet. The other half of the group reported BMI levels of overweight and/or obesity. Based on these data, it is not possible to infer the relation between diet and the increase in BMI. However, previous evidence suggested unsupervised diet is related to increased BMI and waist circumference in individuals who were eutrophic before (unsupervised) diet.^(22)^

Regarding the practice of unsupervised diet, it is observed that, despite little knowledge on nutrition adequacy, there is an intense concern with culturally disseminated nutrients (aspects related to nutrition).^(27)^ Mayes et al., pointed out three aspects in this sense, highlighting the simplification of nutrition science to increase the persuasion of dietary orientation, superficial references that justify ideological views, and the presumption that nutrition is the primary value of food.^(27)^ Such distortions of the nutritional function of foods, allied to the intense level of cognitive restraint, may explain the higher levels in the sub-scale “Guilt for desires or for having yielded to them” of the FCQ-T in the Low-Carb Diet Group. Thus, it is important to raise some questions, such as for whom and for what period the diets with carbohydrates restriction are indicated, the need for specialized professional support, and the level of cognitive restraint and its associations with culturally widespread ideas.

### Relation between Food cravings and Binge Eating

Direct correlation between BE levels and food craving levels was observed in FCQ-T and FCQ-S. A BE episode is usually preceded by the food craving, which, by associating with the physiological effect of food deprivation and emotional factors, weakens the regulation exercised by cognitive control.^(28,29)^Vanderlinden et al., pointed out that negative emotions, physiological state and distorted cognition were the most important previous aspects of BE.^(30)^

When the cultural aspect is brought into question, such as the disclosure of concepts, quantities and different ways of eating, issues related to the nutritional factor (as nutrition proposes), and also to behavior factor (exemplified in cognitive restraint), may be associated with an imbalance between the actual amount consumed and eating attitudes (emotions, thoughts, and feelings) directed at carbohydrates.^(5,28,29)^ In this respect, Anguah et al., demonstrated that after four weeks of carbohydrate restriction, there was a drop in food cravings and an increase by 102% in cognitive restraint.^(5)^

In the present study, on the other hand, the scores of cognitive restraint and carbohydrate restriction did not correlate with food craving (trait and status). Future longitudinal studies can investigate, in a more robust way, causal relations between food cravings, cognitive restraint, and diet, especially those with carbohydrate reduction.

### Study limitations

It is important to point out that the present study has limitations in its interpretations. First, evaluation by means of self-report and self-applied questionnaires can question the reliability of the data collected. Additionally, no sample calculation was performed to determine if the sample allows extrapolating to the general population. Also, due to the variation of the concept of a low-carbohydrate diet, a standardized assessment of food consumption would be desirable to characterize individuals with low carbohydrate consumption.

## CONCLUSION

In this study, we observed that individuals with binge eating associated with the practice of a low-carbohydrate diet have greater cognitive restraint, as well as cognitive restraint directed at carbohydrates, although they do not display greater food craving than those who did not go on this diet.

## Appendix

**Appendix 1:** Cognitive restraint scale items (The Three Factor Eating Questionnaire) adapted to cognitive restraint directed at carbohydrates

1. *I deliberately take small helpings (of carbohydrates) as a means of controlling my weight.*
2. *I consciously hold back at meals (concerning carbohydrates) in order not to weight gain.*
3. *I do not eat some foods (source of carbohydrates) because they make me fat.*
4. *How frequently do you avoid “stocking up” on tempting foods (there are source of carbohydrates)?*
5. *How likely are you consciously to eat less (carbohydrate source foods) than you want?*
6. *On a scale from 1 to 8, in which 1 means no restraint in eating (of carbohydrates) (eating whatever you want, whenever you wan itt) and 8 means total restraint (of carbohydrates), (constantly limiting food intake and never “giving in”), what number would you give yourself?*
