# Supplementary material for: Cognitive restraint directed at carbohydrates in individuals on low-carb diet with binge eating: the role of guilt about food cravings
Source: Einstein (Sao Paulo). 2021 Apr 1;19:eAO5599. doi: 10.31744/einstein_journal/2021AO5599 (PMC8020836; doi:10.31744/einstein_journal/2021AO5599)
Supplement: Translation

# Translation: Restrição cognitiva direcionada aos carboidratos em indivíduos praticantes de dieta *low carb* com compulsão alimentar: o envolvimento da culpa pelos desejos por comida

**Authors:** Jônatas de Oliveira, Maíra Stivaleti Colombarolli, Leandro Silva Figueredo, Táki Athanássios Cordás

**Affiliations:** 1 Brasil Faculdade de Medicina, Universidade de São Paulo, São Paulo, SP, Brasil.; 2 Brasil Universidade de São Paulo, Ribeirão Preto, SP, Brasil.; 3 Brasil Beneficência Portuguesa de São Paulo, São Paulo, SP, Brasil.

**Keywords:** Transtorno da compulsão alimentar, Dieta com restrição de carboidratos, Restrição cognitiva

## Abstract

**Objetivo:**

Avaliar se a dieta com restrição de carboidratos acarreta níveis elevados de desejos intenso por comida em indivíduos com compulsão alimentar.

**Métodos:**

Participaram 146 indivíduos com compulsão alimentar divididos nos Grupos Dieta *Low Carb* (n=48) e Grupo Controle (n=98). Foram utilizados como medidas: Escala de Compulsão Alimentar Periódica, Questionário de Hay, Questionário de Desejos Intensos por Comida – Traço e Estado, Subescala de restrição cognitiva e sua versão adaptada para a restrição cognitiva direcionada aos carboidratos. Foram utilizados testes paramétricos para comparação entre grupos (teste *t* de Student) e o teste de correlação de Pearson para verificar correlações entre variáveis de interesse.

**Resultados:**

Não foram encontradas diferenças entre grupos com e sem prática de dieta em relação ao nível de compulsão alimentar ou ao escore total para desejos intensos por comida. As diferenças encontradas foram os maiores níveis de restrição cognitiva (p=0,01), restrição cognitiva para carboidratos (p=0,01) e subescalas de ‘culpa por causa dos desejos’ (p=0,04) no Grupo Dieta *Low Carb*.

**Conclusão:**

Indivíduos com compulsão alimentar e histórico de dieta com restrição de carboidratos (*low carb*) possuem maior restrição cognitiva direcionada aos carboidratos e associação com atitudes alimentares alteradas (culpa pelos desejos).

## INTRODUÇÃO

A prática de dietas restritivas vem sendo empregada popularmente para perda de peso, incluindo motivadores como a necessidade de mudança no estilo de vida, tratamento de doenças crônicas e padrões de beleza e estética.^(1)^ O emprego de algumas práticas sem acompanhamento profissional envolve pensamentos voltados ao controle alimentar, relacionados a uma “restrição cognitiva” (por exemplo: “Eu não como alguns alimentos porque eles me engordam”).^(2)^ As estratégias comportamentais de emagrecimento disparadas por uma restrição cognitiva, como pular refeições, ficar em jejum, controle de porções e contagem de calorias, estão associadas a atitudes negativas em relação ao corpo.^(1)^

A diminuição do consumo de carboidratos na alimentação tem promovido uma série de nomenclaturas. Dietas em que esse consumo varia dentro da faixa de 30g a 130g ao dia são consideradas *low carb*.^(3)^ Nesse conjunto, existem diversas variações dietéticas possíveis, como o controle do conteúdo calórico, que objetiva o balanço energético negativo, constituindo uma dieta *low carb* hipocalórica. É indicada para o tratamento de obesidade, por causar menor liberação de insulina e maior liberação de glucagon, facilitando a oxidação de gordura e poupando massa magra.^(3)^

Quando a diminuição de carboidratos é severa (quantidade igual ou menor a 20g ao dia), ocorre a promoção do estado de cetose, e a dieta enquadra-se na nomenclatura de dieta cetogênica.^(3)^ Como no exemplo da dieta Atkins, que objetiva um consumo menor que 20g de carboidratos por dia nas duas primeiras semanas.^(3)^

Em relação ao surgimento de um desejo intenso por comida decorrente deste tipo de restrição, Castro et al., demonstraram que uma dieta cetogênica de muito baixa caloria (prescrição supervisionada) não causou o aumento dos desejos.^(4)^ O mesmo foi descrito por Anguah et al., que verificaram diminuição dos desejos intensos por comida e aumento em 102% da restrição cognitiva,^(5)^ao se utilizar uma dieta cetogênica com proporções de 14% de carboidratos, 58% de gordura e 28% de proteínas. Em um outro estudo ensaio clínico randomizado com uso de dieta *low carb* os indivíduos que perderam mais peso tiveram aumento no desejo de alimentos calóricos no sexto mês, porém cediam aos desejos com menor frequência.^(6)^

O desejo intenso por comida é definido como uma sensação de vontade intensa que envolve sensação urgência, com afeto negativo e uma série de pensamentos direcionados de antecipação consumatória, sempre para consumo de uma comida específica.^(7)^ Essa condição abrange aspectos cognitivos, emocionais e neurofisiológicos, além de ser influenciada pelo ambiente e pela disponibilidade do alimento.^(8)^ O aumento do desejo intenso é uma característica comumente observada em indivíduos que apresentam episódios de compulsão alimentar (CA), caracterizada pelo consumo objetivo de uma grande quantidade de alimentos com ocorrência em curto período de tempo, sob a sensação de perda de controle.^(9)^

Considerando a prática de restrição de carboidratos e como ela pode influenciar no aumento de desejos intensos, a literatura apresenta evidências limitadas sobre essa relação em indivíduos com característica de comportamento alimentar compulsivo.

## OBJETIVO

Avaliar se a prática de uma dieta com restrição de carboidratos está relacionada com níveis de desejos intenso mais elevados por comida em indivíduos com compulsão alimentar.

## MÉTODOS

### Participantes

O presente estudo caracteriza-se por um delineamento transversal, realizado com uma amostra de 853 alunos da Universidade de São Paulo (USP). Destes, foi selecionado para a presente pesquisa um recorte de 146 participantes, dos quais 124 (84,9%) eram do sexo feminino. Como critérios de inclusão, foram selecionados indivíduos de ambos os sexos cujos escores na Escala de Compulsão Alimentar Periódica (ECAP) eram indicativos da presença de CA (pontuação >17). Foram excluídos do estudo respondentes que declararam não serem alunos vinculados à USP. Além disso, foram desconsiderados no estudo os alunos de graduação do curso de Nutrição, por estarem mais próximos do conhecimento de adequação nutricional e também pela alta prevalência de risco para transtorno alimentar. Neste estudo, também foram excluídos os que reportaram presença de alguns comportamentos compensatórios inapropriados (vômitos autoinduzidos, laxantes e diuréticos) ou que apresentaram baixo peso, segundo o índice de massa corporal (IMC <18,5kg/m^2^).

### Instrumentos

#### Escala de Compulsão Alimentar Periódica (ECAP)

A ECAP tem sido amplamente utilizada para verificar a presença e a gravidade de sintomas de CA.^(10)^ Possui afirmativas em formato Likert e mensura a frequência de sintomas de CA. A versão empregada foi a traduzida e validada no Brasil.^(11)^ A interpretação dos escores segue as notas de corte, classificando os indivíduos em relação à presença e à gravidade da CA, sendo ausência de compulsão se ≤17 pontos; compulsão moderada se entre 18 e 26 pontos e CA severa se ≥27 pontos. Em estudo para verificar a sensibilidade da escala à presença de transtorno de compulsão alimentar (TCA), o ponto de corte de 17 pontos foi comparado com a *Structured Clinical Interview* (SCID), demonstrando sensibilidade de 97,9%, e a confiabilidade teste-reteste segundo o coeficiente Kappa (0,65) e o Kappa ponderado de 0,66, com alfa de Cronbach de 0,89, demonstrando a adequação de suas características psicométricas na população brasileira.^(12)^

#### Questionário de Hay

Desenvolvido por Phillipa Hay, esse questionário avalia a frequência de comportamentos alimentares inapropriados, como CA, métodos compensatórios e a prática de dieta restritiva nos últimos três meses.^(13)^ Neste estudo, foi utilizada a versão adaptada para o português brasileiro, que apresentou aceitáveis indicadores de confiabilidade, com valor de Kappa de 0,92 no estudo de validação dessa versão. Foram utilizadas, especificamente as questões que avaliam a presença de métodos compensatórios inapropriados e a frequência de episódios de CA.^(14)^

#### Subescala de restrição cognitiva do *Three Factor Eating Questionnaire*

A subescala de restrição cognitiva afere a restrição alimentar com o objetivo de modificar o peso ou a forma corporal^(2)^ e foi proposta em sua versão reduzida com 21 questões.^(15)^ No Brasil, foi adaptada para o português^(16)^ e, posteriormente, validada.^(17)^ A escala é organizada no formato Likert de quatro pontos, para os itens de um a vinte, e de oito pontos para a questão 21. Em relação a interpretação dos escores, os valores mais elevados indicam níveis mais altos de restrição cognitiva. Na presente amostra, os indicadores de confiabilidade desse instrumento foram considerados adequados, com alfa de Cronbach de 0,83.

#### Subescala de restrição cognitiva adaptada para carboidratos

A mesma subescala de restrição cognitiva da *Three Factor Eating Questionnaire*^(15,16)^ foi adaptada, com a inclusão de termos relacionados especificamente à restrição de carboidratos. Essa adaptação foi comunicada e previamente aprovada pelo autor da escala, Dr. Jan Karlsson, da *Örebro University*, Suécia, para atender aos objetivos deste estudo. No cabeçalho do questionário, os participantes receberam instruções do que são algumas comidas fonte de carboidratos. A pontuação e a transformação no escore total foi mantida como na versão original, e não foram utilizadas pontuações de corte, sendo os maiores valores indicativos de maior nível de “restrição direcionada aos carboidratos”. O alfa de Cronbach calculado nessa amostra para escala de restrição cognitiva adaptada para carboidratos foi 0,84, demonstrando índices adequados de confiabilidade. Os itens originais e sua adaptação podem ser consultados no apêndice 1.

#### Questionário de Desejos Intensos por Comida – Traço e Estado

Cepeda-Benito et al., desenvolveram o Questionário de Desejos Intensos por Comida – Traço (QDIC-T) e o Questionário de Desejos Intensos por Comida – Estado (QDIC-E), que combinam dois instrumentos, os quais avaliam diferentes aspectos do desejo intenso por comida: um deles acessa o desejo intenso como elemento constitucional (QDIC-T) e o outro como elemento transitório (QDIC-E).^(8)^ Neste estudo, foram utilizadas as duas versões adaptadas para o português brasileiro com resultados satisfatórios nas análises conduzidas.^(18)^ O alfa de Cronbach calculado nesta amostra foi de 0,94 para QDIC-T e 0,88 para QDIC-E, revelando indicadores de confiabilidade satisfatórios.

#### Questionário de frequência de consumo alimentar

Para avaliação do consumo de comidas que são ricos em carboidratos, foram utilizadas questões de um questionário de frequência de consumo alimentar referente aos últimos três meses. Esse questionário é composto por uma escala Likert de sete pontos, que abrange as frequências de “raramente ou nunca” até “2 ou 3 vezes por semana”. Foram selecionados seis tipos de alimentos (pão de forma, pão francês, chocolate, arroz branco, macarrão e biscoito salgado).^(19)^

#### Questão para identificação da prática de dieta *low carb*

Para identificação da prática de dieta com restrição de carboidratos nos últimos três meses, foi utilizada a seguinte questão: Nos últimos 3 meses você, tentou seguir uma dieta *low carb* evitando alimentos fonte de carboidratos? (as opções de resposta eram “sim”, “não” e “não sei o que é dieta *low carb*”).

## Procedimentos

### Coleta de dados

O recrutamento dos participantes foi realizado por meio da divulgação em grupos específicos de rede social (*Facebook*^®^) e também de *e-mails* institucionais. Aqueles que recebiam *e-mails* também eram convidados para compartilhar o *link* de acesso do questionário on-line em redes sociais, com outros universitários da mesma instituição. Os dados foram coletados entre os meses de julho de 2018 e março de 2019. Todas as informações foram coletadas com utilização de questões estruturadas e escalas de avaliação. Informações de altura e peso foram autorrelatadas. Foram atendidos todos os preceitos éticos dispostos pelo Conselho Nacional de Pesquisa com seres humanos, tendo sido devidamente registrada e aprovada pela instituição responsável (número de aprovação: 2.695.532; CAAE: 88846718.7.0000.0065).

## Análise dos dados

Foram calculados, inicialmente, os índices de confiabilidade das escalas na amostra estudada, utilizando-se o alfa de Cronbach. Em seguida, procedeu-se às análises descritivas dos dados para identificação das medidas de tendência central das variáveis numéricas (médias e desvio-padrão – DP) e frequência absoluta e relativa para as variáveis categóricas. A normalidade das variáveis foi analisada considerando-se os parâmetros de assimetria e curtose de até 2,0 e até 7,0, respectivamente, para verificar distorções na distribuição dos dados, conforme critérios sugeridos por Kim.^(20)^ Após verificação da normalidade na distribuição das variáveis em ambos os grupos, prosseguiu-se à execução dos testes paramétricos, para comparação dos resultados médios entre grupos (teste *t* de Student) e verificar correlações entre as variáveis de interesse (correlação de Pearson). Foram calculados, ainda, os tamanhos de efeito das diferenças entre grupos, por meio do cálculo de d de Cohen. Para as variáveis categóricas, a análise de distribuição e comparação entre grupos foi feita utilizando-se o teste do χ^2^. As análises foram conduzidas no software SPSS, versão 25.

## RESULTADOS

Considerando a amostra de indivíduos com presença de CA (n=146), foram identificados 48 participantes que realizaram a dieta com restrição de carboidratos (Grupo Dieta *Low Carb*) e 98 que não a seguiram (Grupo Controle). A idade dos participantes do Grupo Controle foi, em média, de 22,1 anos (DP±3.25), enquanto no Grupo Dieta *Low Carb,* a média de idade foi de 22,0 anos (DP±3.1), sem diferenças significativas entre os grupos (*t*=0,77; p=0,78). Em relação ao IMC, ambos os grupos foram estatisticamente comparáveis, com IMC médio de 26,5kg/m^2^ (DP±5,4) para o Grupo Controle e de 26,5kg/m^2^ (DP±4,5) para o Grupo Dieta *Low Carb,* sem diferenças entre eles (*t*=-0,04; p=0,96). Adicionalmente, são reportados, na tabela 1, os dados referentes às características de saúde, aos hábitos de vida e aos diagnósticos clínicos e psiquiátricos para ambos os grupos.

**Tabela 1:** Distribuição dos participantes, em função da prática de dieta

	Grupo Controle (n=98) n (%)	Grupo Dieta *Low Carb* (n=48) n (%)
Sexo		
Masculino	18 (18,4)	4 (8,3)
Feminino	80 (81,6)	44 (91,7)
Classificação do IMC		
Eutrófico	47 (48)	26 (54,2)
Sobrepeso	28 (28,6)	12 (25)
Obesidade	23 (23,5)	10 (20,8)
Tabagismo, cigarros por dia		
1-3	4 (4,1)	1 (2,1)
4-5	5 (5,1)	2 (4,2)
>10	2 (2,0)	1 (2,1)
Não fuma	87 (88,8)	44 (91,7)
Consumo de bebida alcoólica, vezes por mês		
1-3	44 (44,9)	18 (37,5)
1	9 (9,2)	5 (10,4)
2	11 (11,2)	7 (14,6)
3	4 (4,1)	3 (6,3)
Não consome bebida alcoólica	30 (30,6)	15 (31,3)
Tipo de alimentação		
Onívoro (consome carnes)	83 (84,7)	45 (93,8)
Vegano	2 (2,0)	0
Vegetariano	13 (13,3)	3 (6,3)
Doenças crônicas		
Hipercolesterolemia	4 (4,1)	2 (4,2)
Hipertensão	1 (1,0)	1 (2,1)
Nenhuma doença crônica	93 (94,9)	45 (93,8)
Diagnóstico de TA		
Anorexia nervosa	2 (2,0)	3 (6,3)
Transtorno de CA	12 (12,2)	6 (12,5)
Nenhum diagnóstico de TA	84 (85,7)	39 (81,3)
Diagnóstico psiquiátrico		
Depressão	5 (5,1)	4 (8,3)
Transtorno bipolar	2 (2,0)	1 (2,1)
Transtorno de ansiedade	20 (20,4)	3 (6,3)
Transtorno de ansiedade e depressão	27 (27,5)	15 (31,2)
Nenhum diagnóstico psiquiátrico	44 (44,9)	25 (52,1)
Episódios de CA		
Duas ou mais vezes por semana	24 (24,5)	8 (16,7)
Uma vez por semana	31 (31,6)	20 (41,7)
Menos que uma vez por semana	33 (33,7)	19 (39,6)
Nenhum episódio	10 (10,2)	1 (2,1)

IMC: índice de massa corporal; TA: transtorno alimentar; CA: compulsão alimentar.

Para identificar a relação entre a prática de dieta com restrição de carboidratos e a presença de sintomas de CA, foram feitas análises comparativas, utilizando como critério essa variável (Tabela 2). Não foram encontradas diferenças entre as médias de pontuação reportadas para sintomas de CA, nem nos escores médios do QDIC-T e do QDIC-E. Dentre as diferenças encontradas, observaram-se níveis mais elevados de restrição cognitiva e restrição cognitiva para carboidratos no Grupo Dieta *Low Carb*, sendo essa diferença significativa e com tamanho de efeito considerado grande (d=1,02 e 1,15, respectivamente). O Grupo Dieta *Low Carb* apresentou, ainda, níveis mais altos de culpa pelos desejos por comida, segundo o QDIC-T (p=0,04; d=0,36), em comparação aos indivíduos do Grupo Controle, com a diferença apresentando um tamanho de efeito moderado.

**Tabela 2:** Comparações da pontuação média nos instrumentos de avaliação, em função da prática de dieta com restrição de carboidratos

	Grupo Controle (n=98)		Grupo Dieta *Low-Carb* (n=48)		t*	gl	Valor de p	d^†^
	Média	Desvio padrão	Média	Desvio padrão				
Compulsão alimentar	23,74	4,84	23,35	5,27	0,43	144	0,66	0,08
Escala de restrição cognitiva	13,31	4,38	17,75	4,35	5,77	144	0,01	1,02
Restrição cognitiva de carboidratos	11,74	3,74	16,00	3,70	6,50	144	0,01	1,15
QDIC-T	160,75	29,59	164,58	27,06	0,75	144	0,45	0,13
Intenções e planos para comer	13,56	3,03	13,52	3,38	0,07	144	0,94	0,01
Antecipação do reforço positivo	21,06	4,76	20,98	4,95	0,09	144	0,92	0,02
Antecipação do alívio de sentimentos negativos	12,15	3,45	12,08	3,71	0,11	144	0,91	0,02
Falta de controle	23,25	6,29	24,38	5,83	1,04	144	0,29	0,18
Pensamentos ou preocupações	24,88	7,40	26,08	6,77	0,95	144	0,34	0,17
Desejo como um estado fisiológico	17,79	3,79	17,54	4,09	0,35	144	0,72	0,06
Emoções presentes nos desejos	18,38	4,34	18,17	4,62	0,27	144	0,78	0,05
Gatilhos desencadeadores	17,64	4,01	18,40	3,71	1,09	144	0,27	0,19
Culpa por desejos ou por ter cedido aos mesmos	12,04	4,08	13,44	3,59	2,01	144	0,04	0,36
QDIC-E	49,15	10,55	49,33	11,30	0,09	144	0,92	0,02
Desejo intenso	10,60	3,20	10,33	2,98	0,48	144	0,62	0,09
Antecipação do reforço positivo	10,06	3,05	10,23	2,58	0,32	144	0,74	0,06
Antecipação do alívio de sentimentos negativos	9,38	2,73	9,75	3,26	0,72	144	0,47	0,13
Falta de controle	10,03	2,70	9,73	2,93	0,61	144	0,53	0,11
Desejo como um estado fisiológico	9,08	3,05	9,29	3,34	0,37	144	0,70	0,07

* estatística do teste *t* de Student; ^†^ tamanho do efeito da diferença expresso por meio do d de Cohen. gl: graus de liberdade; QDIC-T: Questionário de Desejos Intensos por Comida – Traço; QDIC-E: Questionário de Desejos Intensos por Comida – Estado.

Os grupos foram comparados, ainda, conforme a classificação do IMC, observando-se que a presença de pessoas com peso eutrófico e com sobrepeso ou obesidade não diferiram entre os grupos. No Grupo Dieta *Low Carb*, 26 eram eutróficos, e 22 apresentavam sobrepeso ou obesidade. No Grupo Controle, 48 estavam na faixa de peso normal, enquanto 47 apresentam sobrepeso ou obesidade (χ^2^ de 0,16; p=0,68). Complementarmente, foram também analisadas as possíveis relações entre as variáveis de IMC, CA, restrição cognitiva, desejos intensos e restrição de carboidratos. Adicionalmente, foram observadas as relações entre essas variáveis e o consumo de alimentos considerados ricos em carboidratos. Os resultados estão expressos na tabela 3.

**Tabela 3:** Correlação de Pearson dos escores médios entre as variáveis de índice de massa corporal, instrumentos de avaliação e frequência de consumo de comidas ricas em carboidratos (n=146)

	IMC	Compulsão alimentar	Restrição cognitiva	QDIC-T	QDIC-E	Restrição cognitiva de carboidratos
IMC						
Compulsão alimentar	0,20^*^					
Restrição cognitiva	-0,04	-0,08				
QDIC-T	0,11	0,47^†^	-0,03			
QDIC-E	0,10	0,25^†^	-0,11	0,66^†^		
Restrição cognitiva de carboidratos	-0,02	-0,06	0,90^†^	0,02	-0,13	
Chocolate	-0,07	-0,04	-0,12	0,02	0,05	-0,13
Pão de forma	-0,03	0,02	0,01	-0,03	-0,12	-0,06
Arroz	0,03	0,05	-0,33^†^	-0,07	0,03	-0,34^†^
Pão francês	0,12	0,26^†^	-0,13	0,13	0,05	-0,14
Macarrão	-0,04	0,04	-0,14	0,17^*^	0,19^*^	-0,21^†^
Biscoito	0,21^**^	-0,07	-0,06	-0,11	-0,11	-0,09

* p<0,05; ^†^ p<0,01. QDIC-T: Questionário de Desejos Intensos por Comida – Traço; QDIC-E: Questionário de Desejos Intensos por Comida – Estado; IMC: índice de massa corporal.

As análises indicaram relação positiva entre o IMC e sintomas de CA (r=0,20) e consumo de biscoito (r=0,21), com essas magnitudes sendo consideradas fracas. A CA esteve relacionada com aumento fraco a moderado dos desejos nas duas escalas do QDIC-T (r*=*0,47) e QDIC-E 0,25). A restrição cognitiva esteve direta e fortemente relacionada à maior restrição cognitiva direcionada a carboidratos (r=0,90) e moderadamente relacionada a menos consumo de arroz (r=-0,33). A restrição cognitiva de carboidratos, por outro lado, esteve relacionada ao menor consumo de arroz (r=-0,34) e de macarrão (r=-0,21), esta última sendo uma fraca associação.

## DISCUSSÃO

Neste estudo, alguns aspectos do comportamento alimentar foram comparados em indivíduos com CA que relataram ter feito, ou não, a restrição de carboidratos (nomeado dieta *low carb*) nos últimos três meses. Os dados de comparação demonstram que os desejos intensos por comida não se apresentaram maiores naqueles que reportaram a prática da dieta. As diferenças encontradas foram os maiores níveis de restrição cognitiva e restrição cognitiva para carboidratos em praticantes de dieta *low carb*.

### Restrição cognitiva e relação com a prática não supervisionada de dieta

Ao avaliar a restrição cognitiva direcionada a carboidratos, foi possível observar que um menor consumo de arroz e macarrão estiveram diretamente relacionados ao perfil de pensamentos restritivos. A restrição cognitiva pode estar relacionada com atitudes alimentares distorcidas, como no exemplo de uma questão da subescala de restrição cognitiva (“Eu não como alguns alimentos porque eles me engordam”). Em indivíduos com CA, essas atitudes promovem um comer transtornado, pois desconsideram-se o contexto e a frequência em que “algumas comidas” poderiam contribuir para o ganho de peso.^(21)^

Assim, a restrição de carboidratos deve ser discutida como possibilidade de intervenção se ocorrer sob cuidados especializados, por um tempo determinado, com fases de adaptação e avaliações comportamentais.^(4,5)^ Apesar disso, cresce a prevalência da prática não supervisionada de dietas restritivas.^(22-25)^ Hume et al., demonstraram que praticantes de dieta bem-sucedidos relataram menor consumo de carboidratos, maior restrição alimentar e maior consumo proteico e atividade física mais vigorosa.^(26)^

Neste estudo metade do grupo que realizou dieta com restrição de carboidratos reportou peso eutrófico, deixando dúvida sobre o motivador para a prática da dieta. A outra metade do grupo reportou níveis de IMC de sobrepeso e/ou obesidade. Não se pode, a partir desse dado, inferir sobre a relação entre a prática de dieta e o aumento do IMC. Porém, evidências anteriores sugerem que a prática de dieta não supervisionada está relacionada com aumento do IMC e circunferência da cintura em indivíduos que apresentavam peso eutrófico antes da prática da dieta (não supervisionada).^(22)^

Sobre a prática de dieta não supervisionada, observa-se que, apesar do pouco conhecimento sobre adequação nutricional, existe preocupação intensa com nutrientes culturalmente divulgados (aspectos ligados ao nutricionismo).^(27)^ Mayes et al., apontam três aspectos nesse sentido, destacando a simplificação da ciência da nutrição para aumentar a persuasão da orientação dietética, referências superficiais que justificam visões ideológicas e a presunção de que a nutrição é o valor primário da comida.^(27)^ Tais distorções sobre o papel nutricional dos alimentos, aliadas ao intenso nível de restrição cognitiva, podem explicar os maiores níveis na subescala “Culpa por desejos ou por ter cedido aos mesmos” do QDIC-T no Grupo Dieta *Low Carb*. Assim, é importante levantar questões, como para quem e por qual período as dietas com restrição de carboidratos são indicadas, a necessidade de suporte profissional especializado e o nível de restrição cognitiva e suas associações com ideias culturalmente difundidas.

### Relação entre o Desejo intenso por comida e a Compulsão Alimentar

Observou-se correlação direta entre os níveis de CA e níveis de desejo no QDIC-T e no QDIC-E. Um episódio de CA geralmente é precedido pela sensação de um desejo intenso de comer, que, ao se associar com o efeito fisiológico da privação alimentar e fatores emocionais, enfraquece a regulação exercida pelo controle cognitivo.^(28,29)^ Vanderlinden et al., apontaram que emoções negativas, estado fisiológico e cognições distorcidas foram os antecedentes mais importantes da CA.^(30)^

Quando é levado em questão o aspecto cultural, como a divulgação de conceitos, as quantidades e as diferentes formas de comer, questões relacionadas ao fator nutricional (como o nutricionismo propõe) e também ao comportamental, (exemplificada na restrição cognitiva) podem estar associadas com um desbalanço entre a real quantidade consumida e as atitudes alimentares (emoções, pensamentos e sentimentos) direcionadas aos carboidratos.^(5,28,29)^A esse respeito, Anguah et al., demonstraram que, após quatro semanas de restrição de carboidratos, houve diminuição dos desejos intensos por comida e aumento em 102% da restrição cognitiva.^(5)^

No presente estudo, por outro lado, os escores de restrição cognitiva e restrição para carboidratos não apresentaram correlação com os desejos intensos por comida (Traço e Estado). Estudos longitudinais futuros podem investigar, de forma mais robusta, relações causais entre desejos intensos por comida, restrição cognitiva e prática de dieta, especialmente aquelas com redução de carboidratos.

### Limitações do estudo

É preciso pontuar que o presente estudo possui limitações em suas interpretações. Primeiramente, a avaliação por meio de autorrelato e questionários autoaplicáveis pode colocar em questão a fidedignidade dos dados coletados. Além disso, não foi realizado cálculo amostral, para determinar se a amostra permite extrapolação para a população em geral. Adicionalmente, devido à própria variação do conceito de dieta baixa em carboidratos, seria desejável uma avaliação padronizada do consumo alimentar que caracterize os indivíduos em baixo consumo de carboidratos.

## CONCLUSÃO

Nesse estudo, observamos que indivíduos com compulsão alimentar associada com a prática de uma dieta baixa em carboidrato possuem maior restrição cognitiva e restrição cognitiva direcionada aos carboidratos, apesar de não apresentarem maior nível de desejo intenso por comida em relação aos que não fizeram a dieta.

## Apêndice

**Apêndice 1:** Itens da escala de restrição cognitiva (*The Three Factor Eating Questionnaire*) adaptados à restrição cognitiva direcionada a carboidratos

1. Eu deliberadamente consumo pequenas porções (de alimentos fonte de carboidratos) para controlar meu peso./ *I deliberately take small helpings (of carbohydrates) as a means of controlling my weight.*
2. Eu conscientemente me controlo nas refeições (em relação aos carboidratos) para evitar ganhar peso.*/ I consciously hold back at meals (concerning to carbohydrates) in order not to weight gain.*
3. Eu não como alguns alimentos (fonte de carboidratos) porque eles me engordam./ *I do not eat some foods (source of carbohydrates) because they make me fat.*
4. O quanto frequentemente você evita “estocar” (ou se aprovisionar de) comidas tentadoras (que são fonte de carboidratos)?/ *How frequently do you avoid “stocking up” on tempting foods (there are source of carbohydrates)?*
5. O quanto você estaria disposto(a) a fazer um esforço para comer menos (alimentos fonte de carboidratos) do que deseja?/ *How likely are you to consciously eat less (carbohydrate source foods) than you want?*
6. Em uma escala de 1 a 8, onde 1 significa nenhuma restrição alimentar (de carboidratos), e 8 significa restrição total (de carboidratos), qual número você daria para si mesmo?/ *On a scale of 1 to 8, where 1 means no restraint in eating (of carbohydrates) (eating whatever you want, whenever you want it ) and 8 means total restraint (of carbohydrates), (constantly limiting food intake and never “giving in”), what number would you give yourself*?
